# Expanding Primary Care Capacity to Treat Hepatitis C Virus Infection Through an Evidence-Based Care Model — Arizona and Utah, 2012–2014

**Published:** 2014-05-09

**Authors:** Kiren Mitruka, Karla Thornton, Susanne Cusick, Christina Orme, Ann Moore, Richard A. Manch, Terry Box, Christie Carroll, Deborah Holtzman, John W. Ward

**Affiliations:** 1 Division of Viral Hepatitis, National Center for National Center for HIV/AIDS, Viral Hepatitis, STD, and TB Prevention, CDC; 2 University of New Mexico Health Sciences Center; 3 University of Utah School of Medicine; 4 St. Joseph’s Hospital and Medical Center, Phoenix, Arizona

Hepatitis C virus (HCV) infection is the leading reason for liver transplantation and a common cause of hepatocellular carcinoma, the most rapidly increasing cause of cancer-related deaths in the United States (1,2). Of the approximately 3 million persons living with HCV infection in the United States, an estimated 38% are linked to care, 11% are treated, and 6% achieve cure (3). Recent development of highly effective and well-tolerated medications, such as sofosbuvir and simeprevir, to treat chronic HCV infection shows promise in curbing rising HCV-related morbidity and mortality, with the potential to cure >90% of patients. To fully benefit from these new treatments, improvement in linkage to care and treatment is urgently needed.* Lack of provider expertise in HCV treatment and limited access to specialists are well-documented barriers to HCV treatment (4,5). In September 2012, CDC funded programs in Utah and Arizona to improve access to primary care providers with the capacity to manage and treat HCV infection. Both programs were modeled on the Extension for Community Healthcare Outcomes (Project ECHO), developed by the University of New Mexico’s Health Sciences Center in 2003 to build primary care capacity to treat diseases among rural, underserved populations through videoconferencing and case-based learning in “teleECHO” clinics. To assess the effectiveness of these programs in improving primary care provider capacity and increasing the number of patients initiating treatment, process and patient outcome data for each state program were analyzed. In both states, Project ECHO was successfully implemented, training 66 primary care clinicians, predominantly from rural settings. Nearly all (93%) of the clinicians had no prior experience in care and treatment of HCV infection. In both states combined, 129 (46%) of HCV-infected patients seen in teleECHO clinics received antiviral treatment, more than doubling the proportion of patients expected to receive treatment (3). These findings demonstrate Project ECHO’s ability to expand primary care capacity to treat HCV infection, notably among underserved populations.

Project ECHO was designed to build primary care clinicians’ capacity to treat chronic, common, and complex diseases through weekly teleECHO clinics called “Knowledge Networks,” in which primary care clinicians present their cases, through videoconferencing, to specialists who provide advice and clinical mentoring. Working together and supplemented with short didactic presentations (e.g., on HCV diagnosis and management) by interdisciplinary experts, the community-based providers and specialists manage patients following evidence-based protocols.

From September 30, 2012, to February 28, 2014, ECHO programs in Utah and Arizona recruited providers serving populations at increased risk for HCV infection (e.g., persons born during 1945–1965) and in areas with a shortage of HCV specialists. Providers with an interest in treating HCV infection and access to videoconferencing technology (e.g., access to a webcam and software provided by Project ECHO) were eligible to participate. Utah targeted community-based providers in seven neighboring states (Oregon, California, Idaho, Utah, Montana, Wyoming, and Colorado) with an estimated population of 10 million, 60% of whom resided in rural settings. Arizona recruited community-based providers within nine of Arizona’s 15 counties, representing approximately 90% of the state’s population of nearly 7 million.

Utah recruited providers throughout the project period via outreach at professional societies, departments of health, community-based organizations, and university-based referral clinics. Arizona recruited all providers within the first 3 months of the project through outreach at community health centers. In both states, Project ECHO staff initially visited providers to train them in HCV diagnosis and management and in the protocol for patient presentation. Providers then began weekly participation in teleECHO clinic sessions, following the Project ECHO format, and lasting about 1 to 1.5 hours. Providers were eligible to receive continuing medical education credits. Utah’s team of specialists consisted of a hepatologist, psychiatrist, and pharmacist; Arizona’s team consisted of a hepatologist and nurse practitioner. In Utah, teleECHO clinics were held once weekly.

After the initial case presentation, providers were encouraged to attend sessions at specific time intervals (4, 8, 12, 24, and 48 weeks, and 6 months post-treatment) based on standards for monitoring treatment; three to 14 primary care clinicians attended each session (median = six). In Arizona, teleECHO clinics were held once weekly and were site-specific; one to 21 primary care clinicians attended each session (median = seven). Providers at each site were asked to present every patient, those newly diagnosed and those on treatment, at the weekly teleECHO clinic sessions. A monthly synchronous cohort treatment initiation approach was followed, where patients at each site were started on treatment in like timeframes and managed together as a cohort to simplify monitoring. At larger provider sites, an HCV coordinator supported providers in patient management (i.e., medication adherence and insurance enrollment). In Utah, the program collaborated with the local health department to identify HCV-infected patients requiring linkage to care and those who were lost to follow-up. Data from each state Project ECHO program (e.g., types of providers, practice settings, patient characteristics, and clinical outcomes) are summarized in this report.

Over the 17-month period (September 30, 2012–February 28, 2014), a total of 90 unique attendees participated in teleECHO clinics in the two states; of these, 66 (73%) were primary care clinicians with practices in predominantly rural settings and at community health centers (Table 1). A total of 280 unique cases of chronic HCV infection were presented in teleECHO sessions (Table 2). In both states, cases were predominantly among persons who were U.S.-born, non-Hispanic white, and born during 1945–1965. A history of injecting drug use was known for 41.4% (116 of 280) of patients. A total 136 of patients seen were known to have health-care coverage; Medicaid was the most common type of coverage (61.8%) followed by private insurance (23.5%). HCV genotype 1 infection was the most common type of infection (62.9%). Of patients with an available aspartate aminotransferase (AST) to platelet ratio index (APRI)† score, 41% (100 of 243) had a score ≥1, indicating the presence of advanced fibrosis or cirrhosis. Among 129 (46.1% of 280) patients who started treatment, 70.5% (91 of 129) were treated with an interferon-based regimen, and 26.4% (34 of 129) were treated with a regimen containing sofosbuvir, a drug approved in December 2013. Arizona and Utah started treatment with a sofosbuvir-based regimen in 35.8% (29 of 81) and 10.4% (five of 48) of patients, respectively, during December 2013–February 2014.

## Discussion

The implementation of the Project ECHO model in two states demonstrated the utility of this care model in expanding the capacity of primary care clinicians to treat HCV infection. By building collaborations with specialists facilitated by regular videoconferencing, both states recruited and trained clinicians from predominantly rural settings. Almost all (93.9%) of the primary care clinicians had no prior experience in managing HCV infection. Approximately 46% of all patients seen started treatment, a proportion that was more than twice that observed from a CDC study in which 14%–22% of those detected started treatment (3). In a study comparing care delivered by specialists in an HCV clinic at an academic medical center with HCV care and treatment delivered by primary care providers participating in teleECHO clinics, investigators found that care at both settings was equally safe and effective in achieving cure (6). Project ECHO also has been shown to develop knowledge and self-efficacy among participating primary care providers to deliver best-practice care for chronic HCV infection (7).

Each state adapted the Project ECHO model to fit expected needs of its program. In Utah, the health department played an important role in case finding, including those lost to follow up, whereas in Arizona, hepatitis C coordinators were hired to assist clinicians with case management. Arizona also had more frequent presentations (each patient was seen every week), and treatment initiations were synchronized by site.

The findings in this report are subject to at least four limitations. First, treatment completion among some patients who started treatment could not be assessed because patients were either on treatment or had completed therapy and had pending laboratory data at the time of this evaluation. Second, the reasons that treatment was not initiated for some patients could not be assessed. Third, the analysis did not compare differences between Project ECHO implementation in each state and patient treatment decisions. Finally, both Utah and Arizona had either developed a Project ECHO–based program or were in the process of developing it during the 1 year before September 2012; therefore, these state programs might not be representative of programs that might be earlier in development.

CDC and the U.S. Preventive Services Task Force recommend HCV testing for persons born during 1945–1965 and others at risk for HCV infection (8). Studies have revealed that full implementation of these recommendations can avert approximately 120,000 HCV-associated deaths (9). However, limitations in care capacity, particularly in rural areas and other resource-constrained settings, are barriers to achieving the public health benefits of HCV testing, care, and treatment. With training and supervision by specialists, HCV antiviral treatment can be safely and effectively delivered in primary care settings (6,10). Additional safe and effective HCV therapies currently under development could provide new options for primary care clinicians to incorporate management of HCV infection into their practices. Collaborations with specialists will help primary care providers to begin to incorporate new treatments for HCV infection and will be an important measure for improving access and reducing barriers to treatment. The results of this evaluation demonstrate Project ECHO as a model that can enhance primary care provider capacity to treat HCV infection among underserved populations, including the use of newly approved medications.

What is already known on this topic?

In the United States, about 3 million persons are estimated to be living with hepatitis C virus (HCV) infection, which is an important cause of morbidity and mortality. However, there is a documented lack of expertise in HCV-related care and treatment among U.S. primary care providers and limited access to specialists, both of which serve as barriers to life-saving treatment for those who are infected. The Extension for Community Healthcare Outcomes project (Project ECHO) has been shown to be an effective model to overcome these barriers.

What is added by this report?

The Project ECHO model was successfully implemented in two states, training 66 primary care clinicians, predominantly from rural settings. Nearly all (93%) of the clinicians had no prior experience in care and treatment of HCV infection. In both states combined, 46% of HCV-infected patients seen in teleECHO clinics received antiviral treatment, a proportion that was more than twice that observed in a CDC study, further demonstrating the utility of this approach in expanding the capacity of primary care providers to treat HCV infection.

What are the implications for public health practice?

The Project ECHO model is an effective evidence-based model that can be used by state and local areas to enhance capacity to manage and treat HCV infection, especially among underserved populations.

## Figures and Tables

**TABLE 1:** Number and percentage of clinicians participating in Project ECHO case-based learning clinics (teleECHO clinics), by selected characteristics — Arizona and Utah, September 30, 2012–February 28, 2014

	Total		Utah		Arizona	
			
Characteristic	No.	(%)	No.	(%)	No.	(%)
**Total no. of sessions**	**179**		**47**		**132**	
**Total no. of attendees**	**1,722**		**304**		**1,418**	
**No. of unique attendees**	**90**		39		51	
**Occupation of attendees**						
Physician (MD or DO degree)	**44**	**(48.9)**	23	(59.0)	21	(41.2)
Other clinician (RN, PA, or NP degree)	**24**	**(26.7)**	12	(30.8)	12	(23.5)
Pharmacist	**4**	**(4.4)**	2	(5.1)	2	(3.9)
Medical assistant	**12**	**(13.3)**	0	—	12	(23.5)
Students (medical, pharmacy, or nursing)	**4**	**(4.4)**	1	(2.6)	3	(5.9)
Other	**2**	**(2.2)**	1	(2.6)	1	(2.0)
No. of unique primary care clinician attendees*	**66**	**(73.3)**	35	(89.7)	31	(60.8)
**Practice setting of primary care clinicians** * †						
Urban	**15**	**(22.7)**	14	(40.0)	1	(3.2)
Rural	**51**	**(77.3)**	21	(60.0)	30	(96.8)
**Practice type of primary care clinicians** * †						
Community health center (federally qualified health centers)	**32**	**(48.5)**	12	(34.3)	20	(64.5)
Private practice	**8**	**(12.1)**	8	(22.9)	0	—
Hospital-affiliated practice	**16**	**(24.2)**	8	(22.9)	8	(25.8)
Academic medical center	**4**	**(6.1)**	4	(11.4)	0	—
Indian Health Service	**4**	**(6.1)**	3	(8.6)	1	(3.2)
Church-sponsored indigent care clinic	**2**	**(3.0)**	0	—	2	(6.5)
Primary care clinician without prior experience in treating HCV*†	**62**	**(93.3)**	32	(91.4)	30	(96.8)

**Abbreviations:** ECHO = Extension for Community Healthcare Outcomes; HCV = hepatitis C virus.

* With an MD, DO, NP, or PA degree.

† Denominator is the number of unique primary care clinicians.

**TABLE 2:** Number and percentage of HCV-infected patients seen in Project ECHO case-based learning clinics (teleECHO clinics), by selected characteristics — Arizona and Utah, September 30, 2012–February 28, 2014

	Total		Arizona		Utah	
			
Characteristic	No.	(%)	No.	(%)	No.	(%)
**Total no. of patients**	**280**	**(100.0)**	**159**	**(100.0)**	**121**	**(100.0)**
**Birth country**						
U.S.-born	**203**	**(72.5)**	84	(52.8)	119	(98.3)
Foreign-born (Mexico)	**5**	**(1.8)**	3	(1.9)	2	(1.7)
Unknown/missing	**72**	**(25.7)**	72	(45.3)	0	—
**Median age (range) (yrs)**	**55 (17–75)**		55 (17–74)		52.75 (23–75)	
**Birth year**						
Before 1945	**10**	**(3.6)**	8	(5.0)	2	(1.7)
1945–1965	**200**	**(71.4)**	111	(69.8)	89	(73.6)
After 1965	**70**	**(25.0)**	40	(25.2)	30	(24.8)
**Race/Ethnicity**						
Non-Hispanic black	**6**	**(2.1)**	2	(1.3)	4	(3.3)
Non-Hispanic white	**177**	**(63.2)**	75	(47.2)	102	(84.3)
Hispanic	**27**	**(9.6)**	19	(11.9)	8	(6.6)
American Indian/Alaska Native	**15**	**(5.4)**	11	(6.9)	4	(3.3)
Unknown/missing	**55**	**(19.6)**	52	(32.7)	3	(2.5)
**Health insurance**						
Yes	**136**	**(48.6)**	76	(47.8)	60	(49.6)
No	**35**	**(12.5)**	14	(8.8)	21	(17.4)
Unknown/missing	**109**	**(38.9)**	69	(43.4)	40	(33.1)
**Type of health-care coverage** *						
Medicare	**18**	**(13.2)**	15	(19.7)	3	(5.0)
Medicaid	**84**	**(61.8)**	46	(60.5)	38	(63.3)
Private	**32**	**(23.5)**	15	(19.7)	17	(28.3)
Other public	**2**	**(1.5)**	0	—	2	(3.3)
None	**35**	**(25.7)**	14	(18.4)	21	(35.0)
Unknown/missing	**109**	**(80.1)**	69	(90.8)	40	(66.7)
**HCV risk factor**						
Known injection drug use ever	**116**	**(41.4)**	50	(31.4)	66	(54.5)
Known injection drug use within 12 mos	**1**	**(0.4)**	0	—	1	(0.8)
Unknown injection drug use	**164**	**(58.6)**	109	(68.6)	55	(45.5)
Known HIV infection	**3**	**(1.1)**	1	(0.6)	2	(1.7)
**AST to platelet ratio index** †						
<1	**143**	**(51.1)**	96	(60.4)	47	(38.8)
>1	**100**	**(35.7)**	47	(29.6)	53	(43.8)
Unknown/missing	**37**	**(13.2)**	16	(10.1)	21	(17.4)
**Genotype**						
1	**176**	**(62.9)**	94	(59.1)	82	(67.8)
2	**39**	**(13.9)**	20	(12.6)	19	(15.7)
3	**36**	**(12.9)**	20	(12.6)	16	(13.2)
4	**3**	**(1.1)**	2	(1.3)	1	(0.8)
Unknown/missing	**26**	**(9.3)**	23	(14.5)	3	(2.5)
**Started on treatment for HCV infection**	**129**	**(46.1)**	81	(50.9)	48	(39.7)
**Treatment regimen** §						
Pegylated interferon + ribavirin	**30**	**(23.3)**	12	(14.8)	18	(37.5)
Pegylated interferon + ribavirin + telaprevir	**54**	**(41.9)**	39	(48.1)	15	(31.3)
Pegylated interferon + ribavirin + boceprevir	**7**	**(5.4)**	1	(1.2)	6	(12.5)
Sofosbuvir + simeprevir	**6**	**(4.7)**	6	(7.4)	0	—
Sofosbuvir + Pegylated interferon + ribavirin	**18**	**(14.0)**	13	(16.0)	5	(10.4)
Sofosbuvir + ribavirin	**10**	**(7.8)**	10	(12.3)	0	—
Unknown/missing	**4**	**(3.1)**	0	—	4	(8.3)

**Abbreviations:** ECHO = Extension for Community Healthcare Outcomes; HCV = hepatitis C virus; HIV = human immunodeficiency virus; AST = aspartate aminotransferase.

* Denominator is the number of patients with health-care coverage.

† Calculated as (AST [IU/L] / upper limit of normal AST [IU/L]) / platelets [10^9^/L] × 100.

§ Denominator is number of patients who started treatment.

## Footnotes

* Information available at http://www.hcvguidelines.org.

† APRI calculated as (AST [IU/L] / upper limit of normal AST [IU/L]) / platelets [10^9^/L] × 100.
